# Percutaneous Mechanical Aspiration of Left-Sided Intracardiac Masses for Prohibitive Surgical Candidates: A Single-Center Experience

**DOI:** 10.1016/j.jscai.2026.105522

**Published:** 2026-07-28

**Authors:** Haris Patail, Imran Naeem Aziz, Joshua Anapoell, Gabrielle Amar, Joshua Goldberg, Makoto Hibino, David Spielvogel, Faraz Mahmood, Daniel M. Spevack, Srihari S. Naidu, Syed Abbas Haidry, Junichi Shimamura, Hasan Ahmad

**Affiliations:** a Department of Cardiology, Westchester Medical Center, New York; b Division of Translational Research, Rush University Medical College, Chicago, Illinois; c School of Medicine, New York Medical College, New York; d Department of Cardiothoracic Surgery, Weill Cornell Medical College, New York; e Department of Cardiac Surgery, Westchester Medical Center, New York; f Department of Anesthesiology, Westchester Medical Center, New York; g Department of Medicine, Westchester Medical Center, New York

**Keywords:** infective endocarditis, mitral valve masses, percutaneous mechanical aspiration, transseptal

## Abstract

**Background:**

Percutaneous mechanical aspiration (PMA) of intracardiac masses is mostly described for right-sided lesions. Left-sided masses are typically managed medically or surgically, with limited data regarding transcatheter removal. PMA is currently considered off-label; however, it may have advantages to debulk lesions and reduce morbidity in high risk patients.

**Methods:**

We performed an observational study of 7, prohibitive surgical candidates who underwent transseptal PMA of mitral valve (MV) masses. Baseline characteristics, echocardiographic findings, procedural details, and in-hospital and short-term outcomes were collected. The primary procedural efficacy end point included procedural success defined by ≥70% mass removal and procedure-related mortality. The primary composite safety end point included in-hospital mortality, cerebrovascular accident, increased valvular regurgitation, and major structural complications.

**Results:**

A total of 7 patients were included. The mean age was 77.9 ± 6.1 years; 71% were women, and the median predicted Society of Thoracic Surgery mortality score was 19.5%. All patients had echocardiographic evidence of large (>1 cm) left-sided masses involving the MV or adjacent structures. The primary efficacy end point was seen in 100% of cases. The primary composite outcome was seen in 14.3%, driven by worsening MV regurgitation. Procedure and fluoroscopy durations demonstrated a downward trend with increased experience. All patients survived to discharge and 30-day survival was 85.7%, with 1 noncardiac death.

**Conclusions:**

Transseptal PMA is feasible for debulking of MV masses in prohibitive surgical candidates. In this early experience, the technique achieved success with low procedural morbidity and encouraging short-term outcomes. Larger studies are needed to define patient selection criteria and long-term efficacy.

## Introduction

Left-sided cardiac masses, including thrombi, tumors, and vegetations, can accurately be diagnosed due to modern echocardiographic techniques and advanced image quality.^1^ Left-sided masses pose significant challenges, as there is up to a 50% risk of systemic embolization. This risk may be underrepresented due to a lack of routine noninvasive imaging modalities in patients, such as computed tomography or magnetic resonance imaging.^2^ It is well established that left-sided infective endocarditis (IE) is more common than right-sided IE.^3^^,^^4^ Left-sided IE is known to occur more often in an older population and those without pre-existing risk factors such as intravenous drug use (IVDU) or intracardiac devices than right-sided IE.^5^^,^^6^ For native-valve endocarditis associated with the mitral valve (MV), early surgical intervention is recommended (class Ib) for patients with severe valve dysfunction resulting in clinical heart failure, infection complicated by fungi or difficult-to-treat organisms, evidence of heart block due to abscess or penetrating lesions, persistent infection despite 5 to 7 days of antimicrobial therapy, or recurrent systemic emboli.^2^ The American Heart Association further recommends surgery for patients with large vegetations measuring >10 mm (class IIa) and mobile vegetations measuring >10 mm with anterior MV leaflet involvement (class IIb).^2^

While surgery is considered the gold standard for IE with complications, it is important to note that a certain population of patients may not in reality undergo surgical intervention despite presenting with guideline-based surgical indications, due to contraindications or prohibitive surgical risk. Studies report that up to one-quarter of eligible patients do not undergo surgery, most commonly due to poor prognosis, hemodynamic instability, cerebrovascular insult, sepsis, or death prior to intervention; mortality is higher among those managed nonoperatively.^7^^,^^8^ These latter patients are subsequently exposed to higher rates of systemic embolization and persistent bacteremia.^9^^,^^10^ For patients treated conservatively, long-term oral suppressive antibiotic therapy has been used; however, relapse, systemic embolization, and late mortality remain important limitations to consider.^2^^,^^11^

Although emerging data support the use of percutaneous mechanical aspiration (PMA), the existing literature is predominantly limited to the treatment of right-sided IE or intracardiac masses. The Cardiac Lesion Extraction and Aspiration Registry for Infective Endocarditis (CLEAR-IE) registry represents the largest multicenter US database evaluating PMA for right-sided lesions to date and demonstrated high procedural success with a low rate of periprocedural complications.^12^ Prior to the CLEAR-IE registry, evidence consisted mainly of single-center retrospective or observational studies, also restricted to right-sided interventions.^13^ Regarding left-sided vegetations and masses, there is significantly limited data about the role of PMA. Given anatomical and access complexity, higher risk of hemodynamic instability, and systemic embolization, PMA for lesions associated with the MV or aortic valve is not often pursued. A majority of published reports, including case reports or case series, include a wide variety of patient characteristics.^14^, ^15^, ^16^ Owing to extremely limited data and the current off-label use, careful consideration should be taken when patients are referred for left-sided PMA.

In light of this real-world clinical context, we pursued PMA as a therapeutic option in patients who were deemed not candidates for surgical intervention and faced limited alternatives. This approach was undertaken to address an unmet need in a vulnerable population.

## Materials and methods

We conducted a single-center, single-arm observational study of 7 patients who underwent percutaneous, transseptal mechanical aspiration of left-sided intracardiac masses (Central Illustration). Our study includes retrospective data collection, and this article is not associated with a future planned registry. Data were collected from the standard in-hospital electronic medical record system. Baseline characteristics, including patient characteristics, comorbidities, and hospitalization data, were included. Periprocedural and in-hospital outcomes were assessed. Ethical standards were maintained, including patient privacy and autonomy. Verbal consent regarding data collection and storage regarding patient demographic characteristics and procedurals was obtained prior to index procedure. The primary procedural efficacy end point included procedural success without procedural death or significant embolic event. We defined procedural success as the debulking of the intracardiac mass by ≥70%,^12^ estimated by intraoperative 2D- and 3D transesophageal echocardiography (TEE), followed by externalization of the retrieved mass. Percentage of debulking was determined by visual estimation during intraoperative TEE. The primary composite safety end point included in-hospital mortality, cerebrovascular accident, increased valvular regurgitation, and major cardiac structural complications according to the Valvular Academic Research Consortium III definitions.^17^ The secondary composite safety end point included 30-day mortality, cerebrovascular accident, myocardial infarction, increased valvular regurgitation, major cardiac structural complications, readmission, and reintervention. Eligible patients included those who had MV masses with a high predicted risk of complications if treated conservatively. All patients presented with indications for vegetation or mass removal due to either size or location of the lesion, in addition to the presence of embolic phenomenon to the peripheral or cerebral vasculature. Predicted risk of complications included those symptomatic, those with recent embolic events, those requiring infection control, or those at high risk for embolization due to anatomic features. Patients must also have been deemed ineligible for open-heart surgery, and patients who underwent removal of right-sided lesions were excluded. A multidisciplinary heart team formally reviewed all cases with thorough review of the electronic medical record and both invasive and noninvasive testing before proceeding with PMA. In addition to Society of Thoracic Surgeons (STS) mortality risk, clinical presentation and baseline comorbidities such as chronic kidney disease, end-stage renal disease, advanced age, frailty, body mass index, and baseline cardiac comorbidities were highly considered during review of all cases. It is important to note that our surgical program is a high-volume, full mechanical support, and heart transplant program with a high level of surgical expertise, and therefore, the threshold for prohibitive surgical risk was relatively high.

**Central Illustration:**
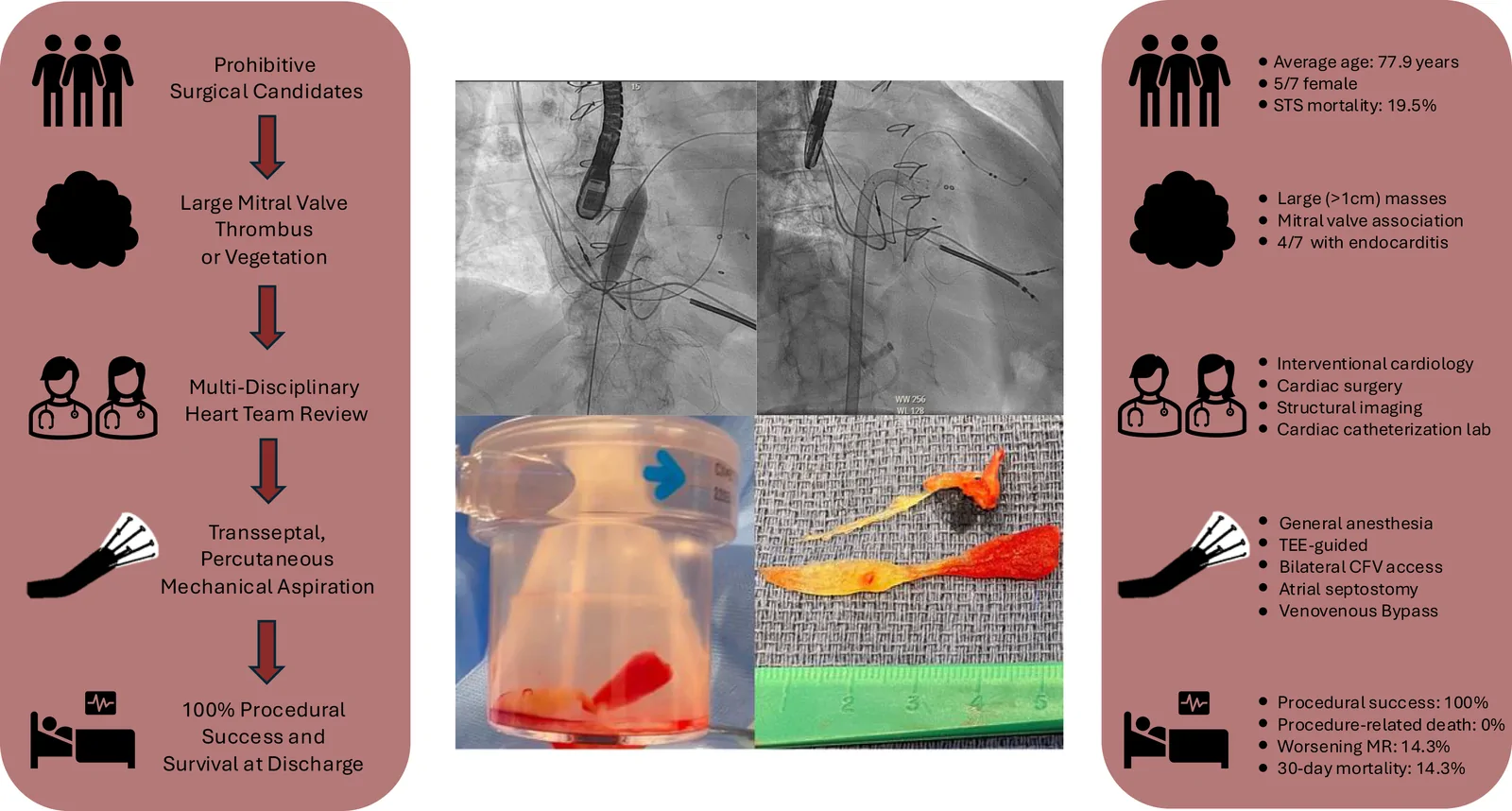
**In asingle-centerstudy, 7 prohibitive surgical candidates underwent multidisciplinary heart team review for percutaneous mechanical aspiration of large,left-sidedintracardiac masses.** CFV, common femoral vein; MR, mitral regurgitation; STS, Society of Thoracic Surgeons; TEE, transesophageal echocardiography.

### Procedural characteristics

**Figure 1:**
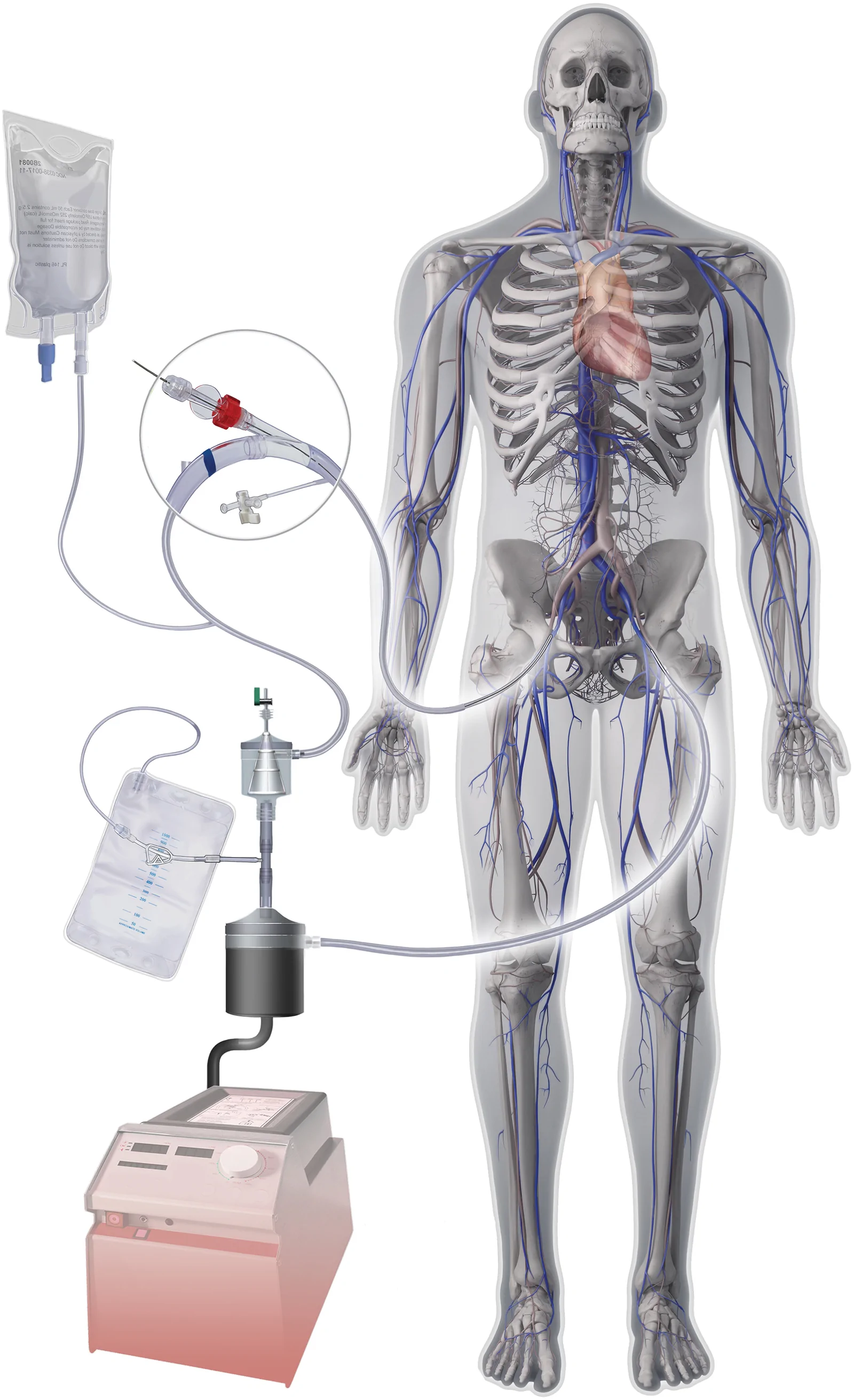
**Bilateral common femoral vein access for both the percutaneous suction cannula (right femoral) and reperfusion cannula (left femoral).** Image courtesy of AngioDynamics Inc and its affiliates.

All patients were placed under general anesthesia in the catheterization laboratory, where they underwent preoperative and intraoperative TEE. The F22^180^ AngioVac (AngioDynamics) cannula was used in all cases. Access included bilateral common femoral vein access in all cases, including a 26F large-bore introducer sheath in the right common femoral vein and a 15- to 17F bypass cannula in the left common femoral vein. Transseptal access was performed via standard atrial septostomy under TEE and fluoroscopic guidance using a 14-mm Armada balloon. SENTINEL Cerebral Protection System (Boston Scientific) was attempted in all cases, with further details of appropriate deployment detailed in the Discussion section. The AngioVac circuit was connected to venovenous bypass with a centrifugal pump and an inline filter for blood reinfusion. An overview of the procedural setup can be viewed in Figure 1.

## Results

Table 1 describes procedural characteristics in detail. A total of 7 patients underwent PMA of MV masses, including masses consistent with endocarditis and noninfected etiologies (Table 2). The mean age was 77.9 ± 6.1 years, and 71.4% (5/7) of patients were women. STS mortality risk was 19.5%, with an IQR of 15% to 23%. Baseline comorbidities, as seen in Table 1, include a majority of patients with hypertension (100%), chronic kidney disease (85.7%), and atrial fibrillation (71.4%). Less than half of the cohort had a history of IVDU (14%), permanent pacemaker or implantable cardioverter-defibrillator (42.8%), intravascular catheter (28.6%), prior sternotomy (14%), and prior endocarditis (0%). Four patients (57%) had prior valve replacements, including surgical, bioprosthetic MV replacement, and transcatheter aortic valve replacement.

**Table 1:** Baseline characteristics and comorbidities

Characteristics	Value
Age, y	77.9 ± 6.1
Body mass index, kg/m^2^	33.1 ± 14.4
STS mortality, %	19.5 (15-23)
Female sex	71.4 (5/7)
Baseline comorbidities	
Intravenous drug use	14.23 (1/7)
Primary pacemaker or implantable defibrillator-cardioverter	42.9 (3/7)
Intravascular catheter	28.6 (2/7)
Hypertension	100 (7/7)
Diabetes mellitus	42.9 (3/7)
Chronic kidney disease	85.7 (6/7)
End-stage renal disease	28.6 (2/7)
Atrial fibrillation	71.4 (5/7)
Coronary artery disease	100 (7/7)
Cardiomyopathy	28.6 (2/7)
History of endocarditis	0 (0/7)
History of sternotomy	14.23 (1/7)
History of valve replacement	57.1 (4/7)

Values are mean ± SD, median (IQR), or % (n/N).

STS, Society of Thoracic Surgeons.

**Table 2:** Baseline mass characteristics and etiology

Patient	Mass etiology	Initial blood culture	Culture data
Patient 1	Thrombus	Negative	No growth
Patient 2	Endocarditis	Positive	Methicillin-sensitive *Staphylococcus aureus*
Patient 3	Endocarditis	Positive	*Streptococcus pyogenes*
Patient 4	Thrombus	Negative	No growth
Patient 5	Endocarditis	Positive	*Streptococcus cristatus*
Patient 6	Endocarditis	Positive	*Streptococcusmitis*
Patient 7	Thrombus	Negative	No growth

All patients had evidence of large (>1 cm) masses or vegetation burden associated with left-sided structures (ie, left atrium, MV, and left ventricle). All patients had evidence of at least mild to moderate mitral regurgitation (MR). Table 3 includes pre- and post-procedure transthoracic echocardiography data, in addition to mass or vegetation location, size, and percent removed.

**Table 3:** Preprocedural and postprocedural transthoracic echocardiography

Patient	EF (%)	Postoperative EF	Mass or vegetation location	Mass or vegetation size, cm	MR severity (preoperative)	MR severity (postoperative)	Mass or vegetation removed, %
Patient 1	60	Unchanged	LA, MV, LV	>1 (multiple)	Mild	Mild	70
Patient 2	60	Unchanged	MV	>2	Mild	Mild	80
Patient 3	60	Unchanged	MV	>1 (multiple)	Moderate	Moderate	80
Patient 4	20	Unchanged	MV	>4	Trivial	Trivial	100
Patient 5	55	Unchanged	Bioprosthetic MV	>2	Mild	Mild	80
Patient 6	65	Unchanged	MV	>1.2	Moderate	Moderate	100
Patient 7	70	Unchanged	LA, MV	>1	Mild	Moderate	90

EF, ejection fraction; LA, left atrium; LV, left ventricle; MR, mitral regurgitation; MV, mitral valve.

**Figure 2:**
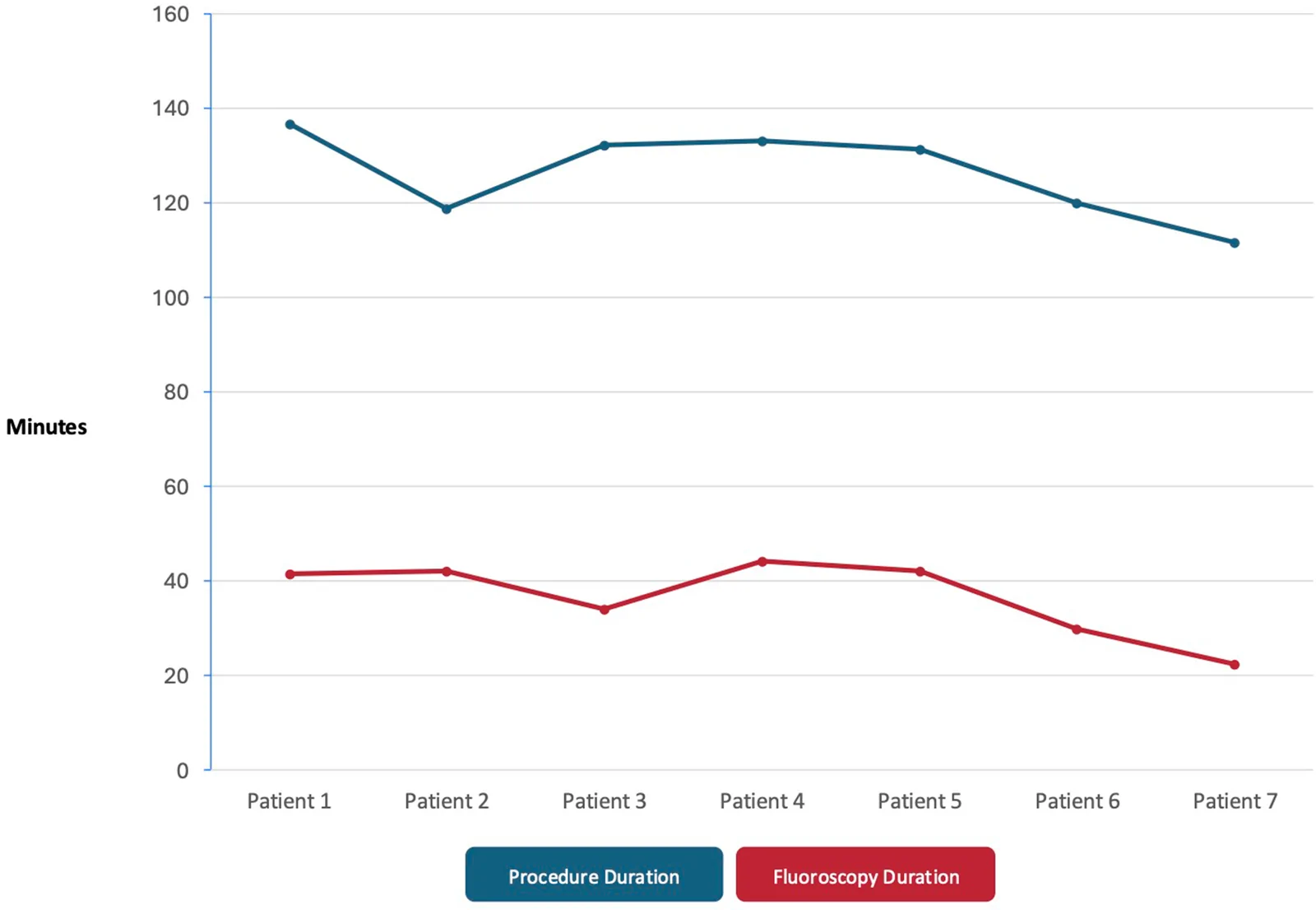
**Procedure and intraprocedural fluoroscopy duration, with a downward trend over time**.

**Figure 3:**
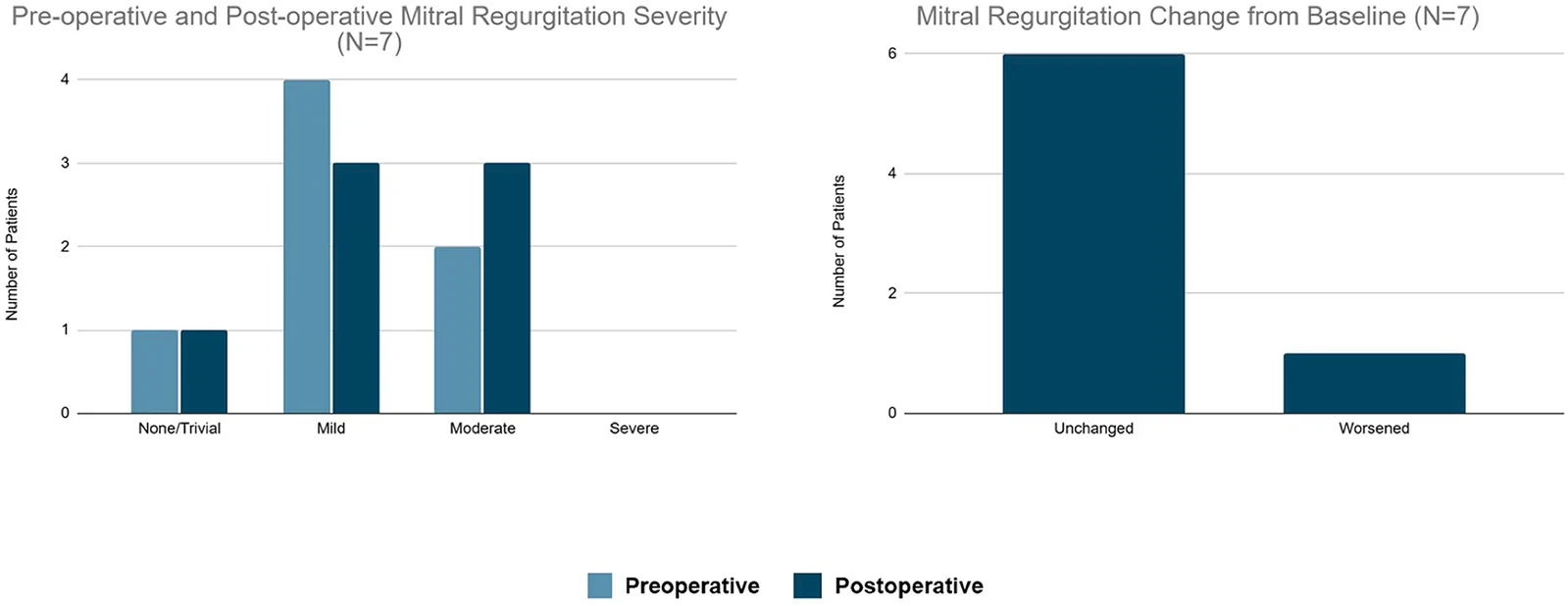
**Differences in preoperative and postoperative mitral regurgitation, in addition to overall changes inmitral regurgitationfrom baseline**.

Preprocedural and postprocedural length of stay varied significantly. The average procedure duration was 2:10 (hours:minutes), and the average fluoroscopy duration was 36.5 ± 7.5 minutes. The average vegetation size removed was 3.02 cm (IQR, 1.9-6.0), and all cases demonstrated ≥70% debulking, while a majority of cases showed even further debulking of >80%. Figure 2 shows the total procedure duration and fluoroscopy time for each patient, both of which decreased with increasing number of procedures. Changes in MR are shown in Figure 3, noting worsening MR in 1 of 7 patients, with a majority of patients showing no changes from baseline valvular function. Additionally, there was no evidence of valve perforation or fulminant valve destruction on postprocedure echocardiography.

Short-term outcomes, described in Table 4, showed that all patients achieved the primary procedural efficacy end point, including both procedural success and freedom from procedure-related death; 14.3% (1/7) of patients sustained the primary composite safety end point, which included in-hospital mortality, cerebrovascular accident, increased valvular regurgitation, and major cardiac structural complications. The primary composite safety end point was driven by worsening valvular function seen in 1 patient. All patients survived at index hospitalization discharge; 6 of 7 patients were alive at 30 days, and 1 patient passed away due to noncardiac causes.

**Table 4:** Procedural and short-term outcomes

Outcome	Value
Preprocedural length of stay, d	8.8 ± 2.8
Postprocedural length of stay, d	18.4 ± 8.5
Procedure duration, h:min	2:10 ± 0.01
Fluoroscopy duration, h:min	36.46 ± 7.5
Average vegetation size removed, cm	3.02 (1.9-6.0)
Percent vegetation removed, %	82 ± 0.11
Postoperative red blood cell transfusion, units	0.4 ± 0.54
Primary procedural efficacy end point	100 (7/7)
Procedural success (>70% vegetation and mass removal)	100 (7/7)
Procedural success without procedure-related death	100 (7/7)
Primary composite safety end point	14.3 (1/7)
In-hospital mortality	0 (0/7)
Cerebrovascular accident	0 (0/7)
Increased valvular regurgitation	14.3 (1/7)
Major cardiac structural complications	0 (0/7)
Secondary composite safety end point	57.1 (4/7)
30-d mortality	14.3 (1/7)
Cerebrovascular accident	0 (0/7)
Myocardial infarction	0 (0/7)
Increased valvular regurgitation	14.3 (1/7)
Readmission	42.9 (3/7)
Major cardiac structural complications	0 (0/7)
Reintervention	0 (0/7)

Values are mean ± SD, median (IQR), or % (n/N).

## Discussion

This study highlights the following key findings: (1) Patients with left-sided cardiac masses and prohibitive surgical risk have an acceptable survival outcome with PMA at 30 days; (2) The procedure can be performed safely by operators with expertise without significantly increasing the risk of stroke, myocardial infarction, or bleeding; and (3) The intervention was successful in achieving significant debulking and/or removal of the vegetation.

To the best of our knowledge, this is the second-largest case series on PMA for the removal of left-sided intracardiac masses in prohibitive surgical candidates.^14^ PMA for left-sided lesions carries inherent risk, including systemic embolization, the complexity of transseptal navigation, and the potential for iatrogenic valvular injury. As a result, published experience with left-sided PMA remains extremely limited, and the available literature consists primarily of isolated case reports and small case series. Compared with the study by Qintar et al,^14^ our cohort was older in age (58 years vs 77 years), in addition to a higher percentage of patients with chronic medical comorbidities such as hypertension, type 2 diabetes mellitus, atrial fibrillation, and renal dysfunction. Similarly, however, both studies demonstrated cohorts with small numbers of IVDU. An important distinguishing feature of our study compared with that of Qintar et al^14^ is the anatomic specificity of the thrombus location. Their cohort included a heterogeneous distribution of thrombi, with 3 cases involving the aortic arch, 3 involving the left atrium or left atrial appendage, and only 3 cases specifically involving the MV. In contrast, our study exclusively evaluated outcomes in patients who underwent this procedure for lesions predominantly centered on the MV. Our cohort, although limited by small sample size, represents patients who underwent PMA for left-sided intracardiac masses due to prohibitive surgical candidacy for MV intervention. These patients reflect a clinical population for whom management options are sharply constrained due to advanced age, high STS surgical mortality risk, comorbidities such as chronic kidney disease and atrial fibrillation, and prior valve interventions. In this setting, based on our single-center experience, PMA offers a potential option for debulking of left-sided masses or vegetations without the morbidity of sternotomy or prolonged postoperative recovery. Notably, less than half of the cohort had a history of IVDU, chronic intravascular catheter requirements, end-stage renal disease, prior endocarditis, and prior sternotomy, which can often be associated with a higher risk of endocarditis in general.^18^ These variables likely had lower representation due to the elderly population, and traditionally, these risk factors are often seen in younger patients who present with right-sided lesions.^5^ As expected with an elderly population (average age of 77 years), a majority of patients had chronic medical comorbidities, including hypertension, chronic kidney disease, and atrial fibrillation. Of 7 patients, 1 had decreased left ventricular systolic function, and 1 patient was particularly a complex case due to a history of bioprosthetic MV replacement, which required PMA for a large, serpiginous MV mass. To our knowledge, there are no prior reports of PMA on a bioprosthetic MV outside of this patient included within our study, which we previously published as a case report on in March 2025.^19^

Within our study, all patients underwent nearly identical procedures, including proceeding with general anesthesia, TEE-guidance, bilateral common femoral vein access, and venovenous bypass with a centrifugal pump with an inline filter for blood reinfusion. Prior cases have documented the use of venoarterial bypass to prevent hemodynamic compromise; however, in our configuration, strictly venous access combined with a transseptal approach and an AngioVac-integrated perfusion circuit enabled continuous mechanical filtration and reperfusion of blood, avoiding arterial cannulation and its associated complications. There were slight differences in bypass cannulation size across patients. Procedure duration was nearly 2 hours per patient, and as seen in Figure 2, there was a decline in overall procedure and fluoroscopy duration as our experience increased within our study. All patients had significant removal of vegetation or mass, including at least 70% in each case, and even more removal in others. Given the significant risk of systemic embolization with left-sided lesions, a SENTINEL cerebral embolic protection device was attempted in all patients; however, deployed in 5 of 7 cases when access and great-vessel anatomy were suitable. Placement was not feasible in patient 4 because of marked great-vessel tortuosity or in patient 7 because of a diminutive right radial artery and left upper-extremity dialysis access. In general, cerebral protection is critical in all cases given the inherent risk of cerebral embolization associated with left-sided lesions. Consistent with prior reports of percutaneous interventions for left-sided IE, cerebral protection was pursued upfront whenever feasible.^14^^,^^15^^,^^20^^,^^21^ Within our study, all patients, regardless of successful cerebral protection placement, were carefully monitored in the cardiac intensive care unit with frequent neurovascular checks over the first 24 hours postoperatively.

All patients survived the index PMA procedure and total hospital stay. Additionally, all patients achieved the primary efficacy end point, including procedural success and freedom from procedure-associated death. All patients were free from in-hospital mortality and acute neurologic or cardiac major events. Major clinical bleeding was low, which was evident by a low average of blood transfusion requirements postoperative, indicated by <1 unit of packed red blood cells per patient. Given a limited sample size and significant heterogeneity, preprocedural and postprocedural length of stay was variable due to ongoing clinical variables unrelated to the index PMA procedure. This underscores the complexity of this patient population, in whom PMA is considered only after multidisciplinary consensus and typically in the setting of advanced age, frailty, or significant hemodynamic or anatomic risk precluding surgery. All patients were discharged, and all except 1 patient demonstrated 1-month survival, who was deceased before 1-month follow-up and was noted to have a noncardiac cause of death. All patients prior to the index procedure had evidence of at least mild MR. When evaluating patients for PMA, careful consideration should be given to native MV structural abnormalities and evidence of valvular damage. Owing to the inherent nature of PMA, which involves a suction mechanism for lesion removal, further damage to the MV or worsening of previously noted valvular regurgitation is a potential risk. In our study, a majority of patients had intact valvular structure without evidence of significant valve destruction, perforation, or severe regurgitation before proceeding with PMA. Only 1 of 7 patients was found to have worsening of MR following the procedure. Worsening MR in this patient was likely due to pre-existing posterior leaflet perforation seen prior to PMA, which was likely exacerbated during the index procedure. Regarding follow-up at 30 days, ∼40% of patients were readmitted; however, causes of readmission included exacerbation of chronic atrial fibrillation, electrolyte derangements due to chronic renal dysfunction, and severe allergic reaction to oral antibiotics, which were not deemed to be directly associated with the index procedure. One patient died at an outside institution prior to completing 1-month follow-up, and it was found that the patient had progressive kidney dysfunction, refused dialysis, and elected for hospice care. To our knowledge, there were no readmissions or deaths necessarily related to the PMA itself (ie, stroke, embolic phenomenon, major bleeding, or structural or vascular complications).

Given the overall paucity of data for PMA, particularly for left-sided lesions, the question remains whether PMA should be offered for patients who are deemed prohibitive surgical candidates. Despite limitations within the literature, more data are emerging for right-sided lesions, including several retrospective studies and the CLEAR-IE registry.^12^^,^^13^ Within the meta-analysis for right-sided masses conducted by Mhanna et al,^13^ procedural success, defined by appropriate debulking, was 89.2%, while clinical success, defined by residual vegetation, was 79.1%. As this meta-analysis assessed >300 patients, the overall survival rate was ∼90% across 44 independent studies.^13^ Regarding patients requiring left-sided PMA, case reports and case series have demonstrated mixed outcomes of procedural success and survival^14^^,^^16^^,^^20^^,^^21^; however, further data are still necessary to determine a true morbidity and mortality benefit.

In real-world practice, ∼19% to 36% of patients with left-sided IE and a surgical indication may not ultimately undergo surgery for numerous reasons.^7^^,^^8^^,^^22^, ^23^, ^24^, ^25^ Patients with left-sided IE who have a surgical indication but are deemed unsuitable for surgery have poor outcomes, with limited options beyond antimicrobial therapy and supportive care, leaving them at high risk for uncontrolled infection, persistent bacteremia, heart failure, and thromboembolic complications.^7^^,^^8^^,^^22^, ^23^, ^24^, ^25^

When considering PMA for patients with left-sided IE, it is important to recognize the poor prognosis of those who have a surgical indication but receive no intervention. Among patients with an identified contraindication to surgery, reported mortality rates range from ∼55% to 78% at 6 months and from 56% to as high as 85% at 1 year.^7^^,^^8^^,^^22^, ^23^, ^24^, ^25^ In a cohort of 42 patients with a surgical indication and contraindication to surgery, 1-year mortality varied according to the indication for surgery: 85.9% among those with heart failure, 76.7% among those with uncontrolled infection, and 72.7% among those with embolic risk.^22^ It is also important to acknowledge that the poor outcomes, at least in part may also be due to a higher baseline risk from comorbidities that disqualified them from the surgery in the first place.

### Limitations

First, this is a single-arm, nonrandomized, retrospective study, and unknown confounders may have influenced our results. These patients were carefully selected by our structural heart team, which may have introduced selection bias. Our sample size was limited to 7 patients, which limits the generalizability of our findings. Most of our cases were native-valve endocarditis, and hence, this level of procedural success may not be seen in patients of prosthetic-valve endocarditis. The majority of our patients were elderly and not IVDU; therefore, it remains unclear if left-sided PMA is equally efficacious and safe for younger patients with recurrent IE due to IVDU.

While ≥70% mass removal is originally derived from right-sided PMA aspiration data, this threshold is generally not validated for PMA in general, and particularly, there are little to no data on this cutoff for left-sided cases. Currently, there is no lesion-specific or side-specific benchmarks that exist in the literature. The use of 70% removal was rationalized because the CLEAR-IE registry is the largest and most rigorous to date for PMA for intracardiac masses in general. This poses a large limitation and challenge for patients with left-sided lesions who are prohibitive surgical candidates, as there currently is no cutoff that correlates with decreased embolic events.

## Conclusions

Transseptal PMA is a complex multidisciplinary procedural approach that can be a feasible and potentially safe method for debulking and partial removal of left-sided intracardiac masses, including vegetation and noninfected masses, in patients who are deemed to be prohibitive surgical candidates. In this early experience, the technique achieved substantial mass debulking with low procedural morbidity and encouraging short-term outcomes. The ability to achieve meaningful procedural success through a percutaneous, transseptal approach may provide a potential therapeutic option for a population of patients with limited options. These findings should be interpreted cautiously given the study’s small sample size, single-center design, lack of a comparator group, and inherent design limitations. Larger studies are needed to define patient selection criteria and long-term efficacy further.

## CRediT authorship contribution statement

**Haris Patail:** Writing – review & editing, Writing – original draft, Visualization, Validation, Supervision, Resources, Project administration, Methodology, Investigation, Formal analysis, Data curation, Conceptualization. **Imran Naeem Aziz:** Writing – review & editing, Writing – original draft, Visualization, Validation, Supervision, Resources, Project administration, Methodology, Investigation, Formal analysis, Data curation, Conceptualization. **Joshua Anapoell:** Writing – review & editing, Writing – original draft, Formal analysis, Data curation. **Gabrielle Amar:** Writing – review & editing, Writing – original draft, Data curation. **Joshua Goldberg:** Writing – review & editing, Visualization, Validation, Supervision, Resources, Project administration, Methodology, Investigation, Formal analysis, Data curation, Conceptualization. **Makoto Hibino:** Writing – review & editing, Visualization, Validation, Resources, Methodology, Investigation, Data curation, Conceptualization. **David Spielvogel:** Writing – review & editing, Project administration, Methodology, Investigation, Data curation, Conceptualization. **Faraz Mahmood:** Writing – review & editing, Writing – original draft, Resources, Methodology, Conceptualization. **Daniel M. Spevack:** Writing – review & editing, Writing – original draft, Visualization, Validation, Supervision, Resources, Project administration, Methodology, Investigation, Formal analysis, Data curation, Conceptualization. **Srihari S. Naidu:** Writing – review & editing, Writing – original draft, Visualization, Validation, Supervision, Resources, Project administration, Methodology, Investigation, Formal analysis, Data curation, Conceptualization. **Syed Abbas Haidry:** Writing – review & editing, Writing – original draft, Visualization, Validation, Supervision, Resources, Project administration, Methodology, Investigation, Formal analysis, Data curation, Conceptualization. **Junichi Shimamura:** Writing – review & editing, Writing – original draft, Visualization, Validation, Supervision, Resources, Project administration, Methodology, Investigation, Formal analysis, Data curation, Conceptualization. **Hasan Ahmad:** Writing – review & editing, Writing – original draft, Visualization, Validation, Supervision, Resources, Project administration, Methodology, Investigation, Formal analysis, Data curation, Conceptualization.

## Declaration of competing interest

The authors declared no potential conflicts of interest with respect to the research, authorship, and/or publication of this article.

## References

[1] Kurmann R., El-Am E., Ahmad A. (2023). Cardiac masses discovered by echocardiogram; what to do next?. Struct Heart.

[2] Baddour L.M., Wilson W.R., Bayer A.S. (2015). Infective endocarditis in adults: diagnosis, antimicrobial therapy, and management of complications: a scientific statement for healthcare professionals from the American Heart Association. Circulation.

[3] Lee Y., Kim J.H., Lee J.A. (2024). Clinical characteristics and risk factors for right-sided infective endocarditis in Korea: a 12-year retrospective cohort study. Sci Rep.

[4] Lassen H., Nielsen S.L., Gill S.U.A., Johansen I.S. (2020). The epidemiology of infective endocarditis with focus on non-device related right-sided infective endocarditis: a retrospective register-based study in the region of Southern Denmark. Int J Infect Dis.

[5] Shmueli H., Thomas F., Flint N., Setia G., Janjic A., Siegel R.J. (2020). Right-sided infective endocarditis 2020: challenges and updates in diagnosis and treatment. J Am Heart Assoc.

[6] Clarelin A., Rasmussen M., Olaison L., Ragnarsson S. (2021). Comparing right- and left sided injection-drug related infective endocarditis. Sci Rep.

[7] Iung B., Doco-Lecompte T., Chocron S. (2016). Cardiac surgery during the acute phase of infective endocarditis: discrepancies between European Society of Cardiology guidelines and practices. Eur Heart J.

[8] Chu V.H., Park L.P., Athan E. (2015). Association between surgical indications, operative risk, and clinical outcome in infective endocarditis: a prospective study from the International Collaboration on Endocarditis. Circulation.

[9] Suruagy-Motta R.F.O., Almeida L.G.S., Tirelli-Rocha J. (2025). Percutaneous mechanical aspiration versus surgical management of tricuspid valve endocarditis: a systematic review and updated meta-analysis. Am J Cardiol.

[10] Pericàs J.M., Llopis J., Athan E. (2021). Prospective cohort study of infective endocarditis in people who inject drugs. J Am Coll Cardiol.

[11] Vallejo C.N., Mateu L., Cediel G. (2021). Long-term antibiotic therapy in patients with surgery-indicated not undergoing surgery infective endocarditis. Cardiol J.

[12] El Sabbagh A., Hibbert B., Bangalore S. (2025). Outcomes of percutaneous mechanical aspiration in right-sided infective endocarditis: a multicenter registry. J Am Coll Cardiol.

[13] Mhanna M., Beran A., Al-Abdouh A. (2022). AngioVac for vegetation debulking in right-sided infective endocarditis: a systematic review and meta-analysis. Curr Probl Cardiol.

[14] Qintar M., Wang D.D., Lee J. (2022). Transcatheter vacuum-assisted left-sided mass extraction with the AngioVac system. Catheter Cardiovasc Interv.

[15] Fiocco A., Colli A., Besola L. (2023). Case report: treatment of left-sided valve endocarditis using the Transapical AngioVac System and cerebral embolism protection device: a case series. Front Cardiovasc Med.

[16] Srivastava S., Al Hennawi H., Athamnah F., Abulshamat A., Bandi A., Qintar M. (2024). Aspiration of left atrial masses using the large-bore manual aspiration system: the ASPIRATE LA procedure. JACC Case Rep.

[17] Stone G.W., Adams D.H., Abraham W.T. (2015). Clinical trial design principles and endpoint definitions for transcatheter mitral valve repair and replacement: part 2: endpoint definitions: a consensus document from the Mitral Valve Academic Research Consortium. J Am Coll Cardiol.

[18] Yallowitz A.W., Decker L.C. (2023).

[19] Amar G., Patail H., Spevack D. (2025). Transcatheter vacuum-assisted mass extraction on a mitral bioprosthesis. JACC Case Rep.

[20] Palatnic L., Manion C., Zlotnick D.M. (2026). Percutaneous debulking of left atrial appendage occlusion device-associated thrombi and vegetation. JACC Case Rep.

[21] Al Asmari A., Chan K.L. (2025). Percutaneous debulking of bioprosthetic mitral valve vegetations: preserving valve function in recurrent infective endocarditis. JACC Case Rep.

[22] Van Hemelrijck M., Sromicki J., Frank M. (2023). Dismal prognosis of patients with operative indication without surgical intervention in active left-sided infective endocarditis. Front Cardiovasc Med.

[23] Habib G., Erba P.A., Iung B. (2019). Clinical presentation, aetiology and outcome of infective endocarditis. Results of the ESC-EORP EURO-ENDO (European infective endocarditis) registry: a prospective cohort study. Eur Heart J.

[24] Carino D., Fernández-Cisneros A., Hernández-Meneses M. (2020). The fate of active left-side infective endocarditis with operative indication in absence of valve surgery. J Card Surg.

[25] Ramos-Martínez A., Calderón-Parra J., Miró J.M. (2019). Effect of the type of surgical indication on mortality in patients with infective endocarditis who are rejected for surgical intervention. Int J Cardiol.

